# Supplementary material for: Anti-CAR Immunity Drives Acquired Therapeutic Resistance to GD2-CAR T Cell Therapy in Diffuse Midline Glioma
Source: medRxiv. 2026 Jul 9:2026.06.25.26356492. Preprint. [Version 1] doi: 10.64898/2026.06.25.26356492 (PMC13370534; doi:10.64898/2026.06.25.26356492)
Supplement: Supplementary file 1 [file media-1.pdf]

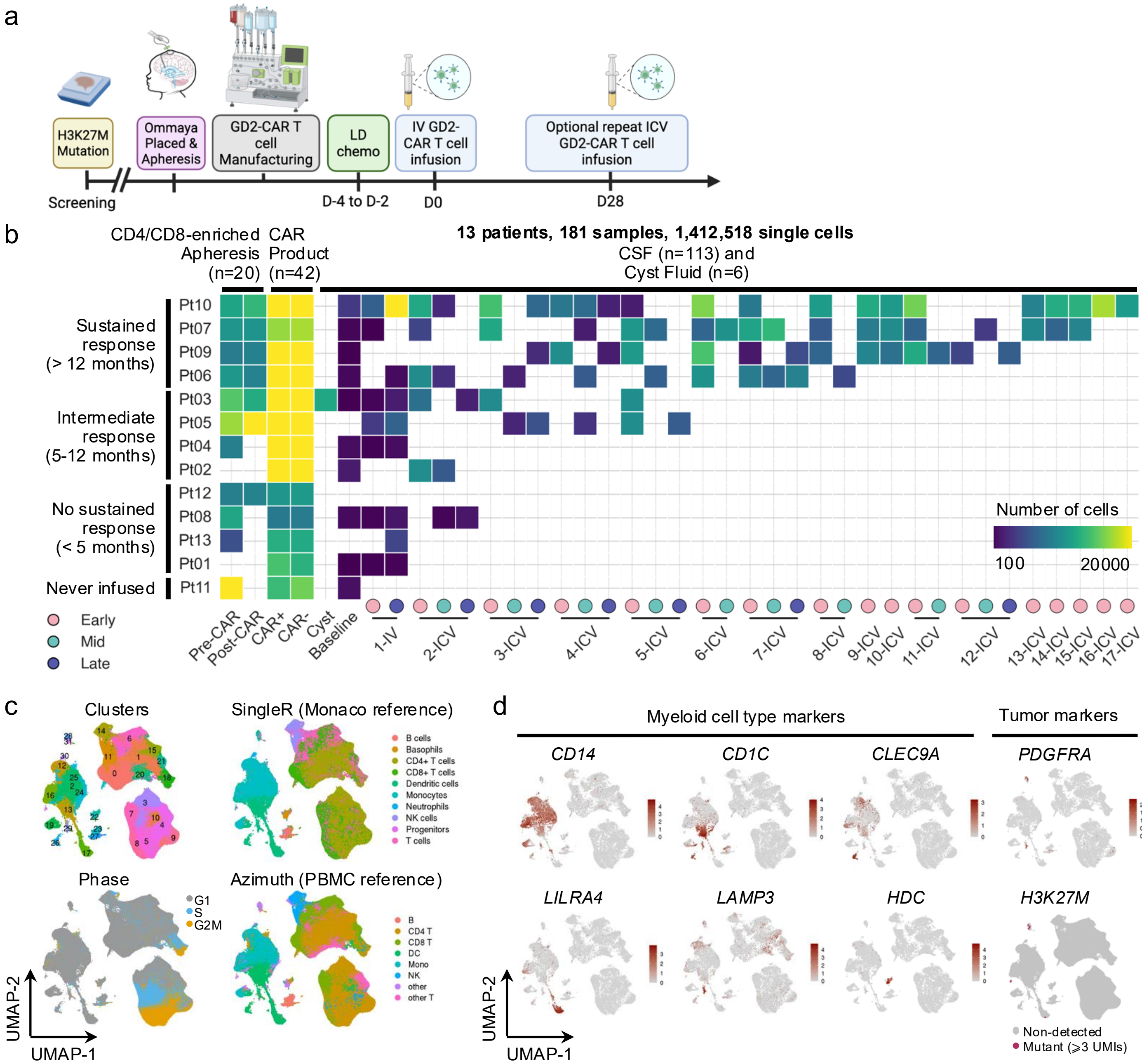
a
H3K27M Mutation
Ommaya Placed & Apheresis
GD2-CAR T cell Manufacturing
LD chemo
IV GD2-CAR T cell infusion
Optional repeat ICV GD2-CAR T cell infusion
Screening
D-4 to D-2
D0
D28
b
CD4/CD8-enriched Apheresis (n=20)
CAR Product (n=42)
13 patients, 181 samples, 1,412,518 single cells
CSF (n=113) and Cyst Fluid (n=6)
Sustained response (> 12 months)
Intermediate response (5-12 months)
No sustained response (< 5 months)
Never infused
Pt10
Pt07
Pt09
Pt06
Pt03
Pt05
Pt04
Pt02
Pt12
Pt08
Pt13
Pt01
Pt11
Number of cells
100
20000
Early
Mid
Late
Pre-CAR
Post-CAR
CAR+
CAR-
Cyst
Baseline
1-IV
2-ICV
3-ICV
4-ICV
5-ICV
6-ICV
7-ICV
8-ICV
9-ICV
10-ICV
11-ICV
12-ICV
13-ICV
14-ICV
15-ICV
16-ICV
17-ICV
c
Clusters
SingleR (Monaco reference)
B cells
Basophils
CD4+ T cells
CD8+ T cells
Dendritic cells
Monocytes
Neutrophils
NK cells
Progenitors
T cells
Phase
G1
S
G2M
Azimuth (PBMC reference)
B
CD4 T
CD8 T
DC
Mono
NK
other
other T
UMAP-2
UMAP-1
d
Myeloid cell type markers
Tumor markers
CD14
CD1C
CLEC9A
PDGFRA
LILRA4
LAMP3
HDC
H3K27M
Non-detected
Mutant (⩾3 UMIs)
UMAP-2
UMAP-1
