# Supplementary material for: Anti-CAR Immunity Drives Acquired Therapeutic Resistance to GD2-CAR T Cell Therapy in Diffuse Midline Glioma
Source: medRxiv. 2026 Jul 9:2026.06.25.26356492. Preprint. [Version 1] doi: 10.64898/2026.06.25.26356492 (PMC13370534; doi:10.64898/2026.06.25.26356492)
Supplement: Supplementary file 2 [file media-10.pdf]

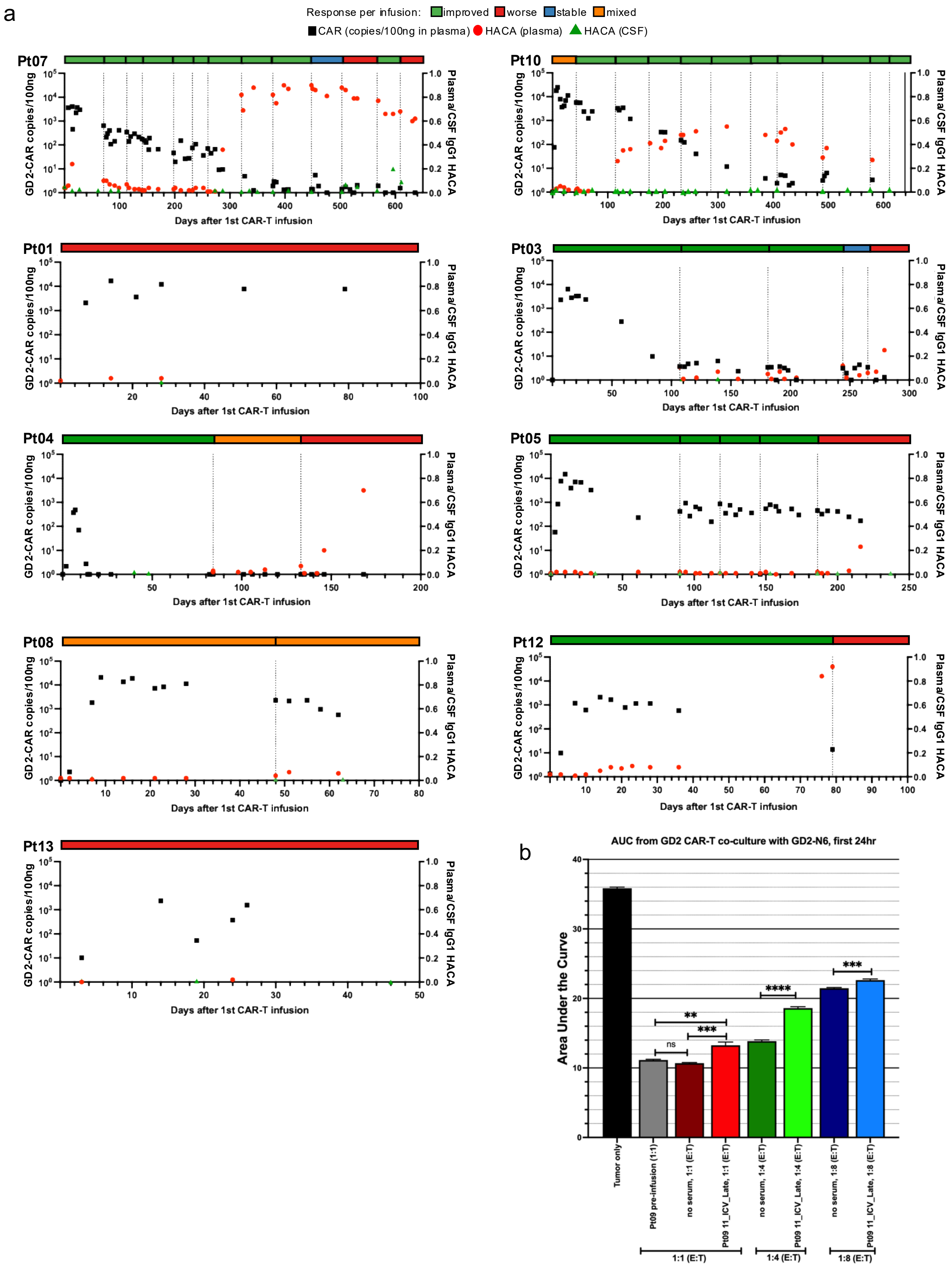
a
Response per infusion: improved worse stable mixed
CAR (copies/100ng in plasma) HACA (plasma) HACA (CSF)
Pt07
Pt10
Pt01
Pt03
Pt04
Pt05
Pt08
Pt12
Pt13
GD2-CAR copies/100ng
Plasma/CSF IgG1 HACA
Days after 1st CAR-T infusion
b
AUC from GD2 CAR-T co-culture with GD2-N6, first 24hr
Area Under the Curve
Tumor only
Pt09 pre-infusion (1:1)
no serum, 1:1 (E:T)
Pt09 11_ICV_Late, 1:1 (E:T)
no serum, 1:4 (E:T)
Pt09 11_ICV_Late, 1:4 (E:T)
no serum, 1:8 (E:T)
Pt09 11_ICV_Late, 1:8 (E:T)
1:1 (E:T)
1:4 (E:T)
1:8 (E:T)
ns
**
***
****
