# Supplementary material for: Anti-CAR Immunity Drives Acquired Therapeutic Resistance to GD2-CAR T Cell Therapy in Diffuse Midline Glioma
Source: medRxiv. 2026 Jul 9:2026.06.25.26356492. Preprint. [Version 1] doi: 10.64898/2026.06.25.26356492 (PMC13370534; doi:10.64898/2026.06.25.26356492)
Supplement: Supplementary file 3 [file media-11.pdf]

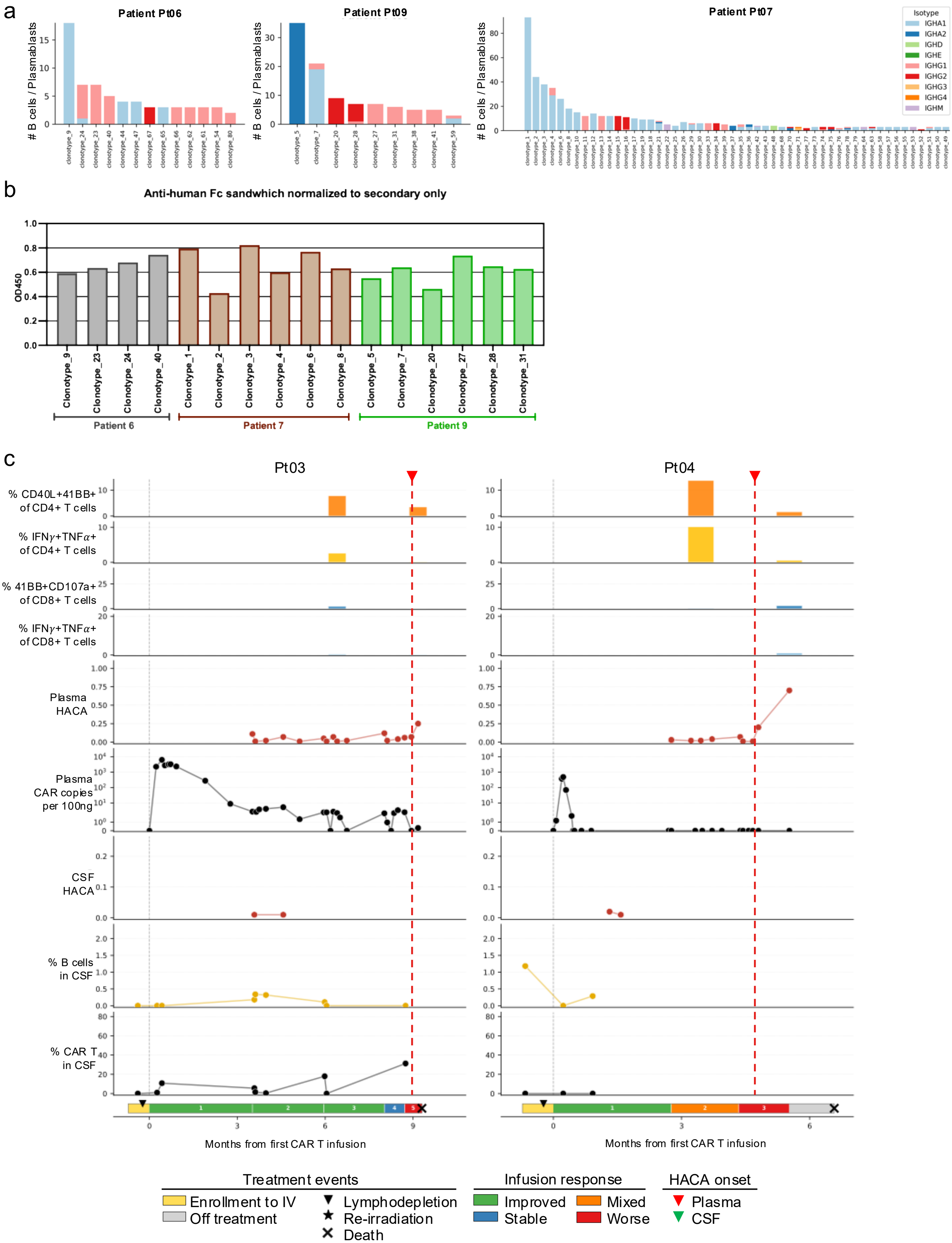
a
Patient Pt06
Patient Pt09
Patient Pt07
# B cells / Plasmablasts
Isotype
IGHA1
IGHA2
IGHD
IGHE
IGHG1
IGHG2
IGHG3
IGHG4
IGHM
b
Anti-human Fc sandwhich normalized to secondary only
OD450
Clonotype_9
Clonotype_23
Clonotype_24
Clonotype_40
Clonotype_1
Clonotype_2
Clonotype_3
Clonotype_4
Clonotype_6
Clonotype_8
Clonotype_5
Clonotype_7
Clonotype_20
Clonotype_27
Clonotype_28
Clonotype_31
Patient 6
Patient 7
Patient 9
c
Pt03
Pt04
% CD40L+41BB+ of CD4+ T cells
% IFNγ+TNFα+ of CD4+ T cells
% 41BB+CD107a+ of CD8+ T cells
% IFNγ+TNFα+ of CD8+ T cells
Plasma HACA
Plasma CAR copies per 100ng
CSF HACA
% B cells in CSF
% CAR T in CSF
Months from first CAR T infusion
Treatment events
Enrollment to IV
Off treatment
Lymphodepletion
Re-irradiation
Death
Infusion response
Improved
Stable
Mixed
Worse
HACA onset
Plasma
CSF
