# Supplementary material for: Anti-CAR Immunity Drives Acquired Therapeutic Resistance to GD2-CAR T Cell Therapy in Diffuse Midline Glioma
Source: medRxiv. 2026 Jul 9:2026.06.25.26356492. Preprint. [Version 1] doi: 10.64898/2026.06.25.26356492 (PMC13370534; doi:10.64898/2026.06.25.26356492)
Supplement: Supplementary file 4 [file media-4.pdf]

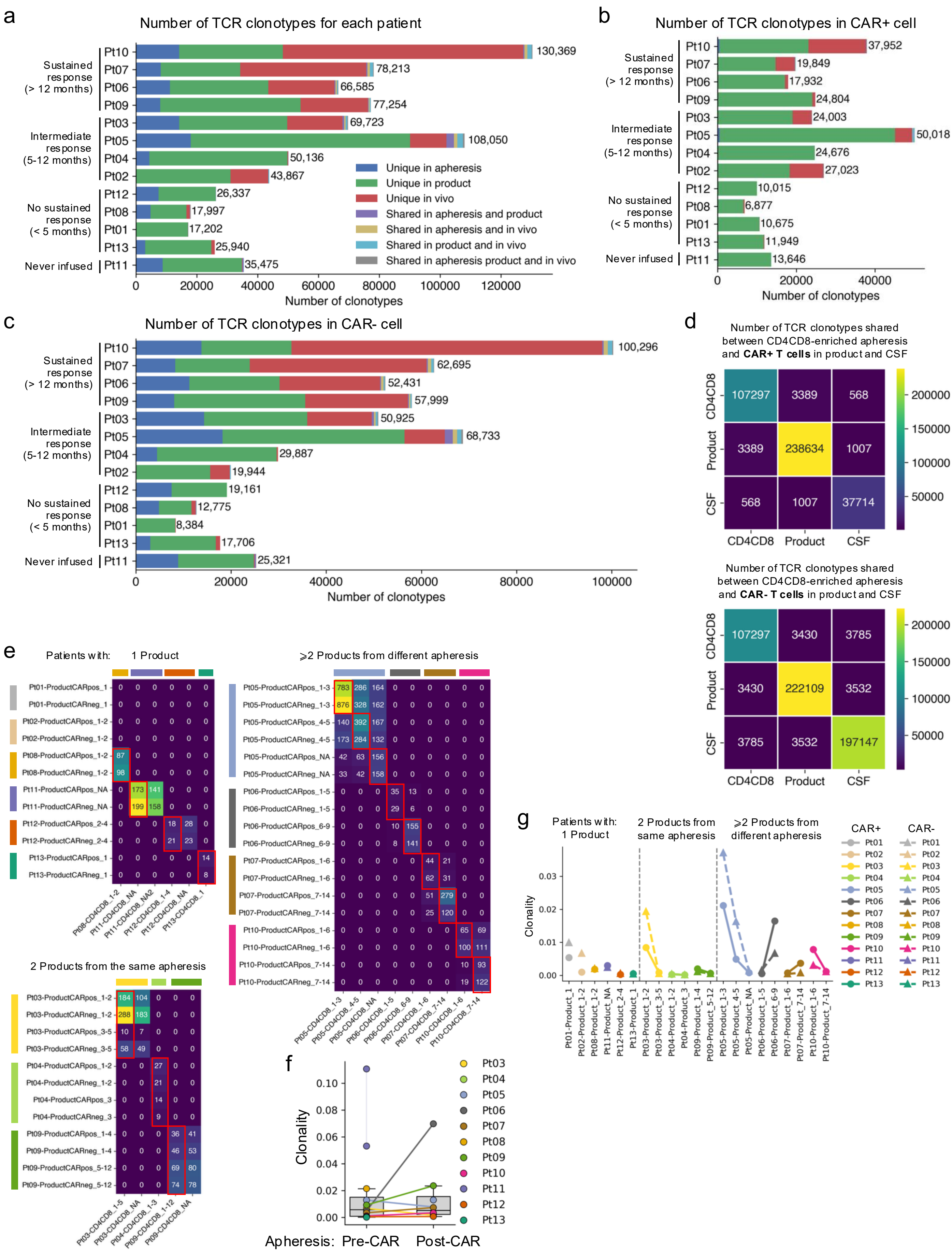

a
Number of TCR clonotypes for each patient
Sustained response (> 12 months)
Intermediate response (5-12 months)
No sustained response (< 5 months)
Never infused
Pt10 130,369
Pt07 78,213
Pt06 66,585
Pt09 77,254
Pt03 69,723
Pt05 108,050
Pt04 50,136
Pt02 43,867
Pt12 26,337
Pt08 17,997
Pt01 17,202
Pt13 25,940
Pt11 35,475
Unique in apheresis
Unique in product
Unique in vivo
Shared in apheresis and product
Shared in apheresis and in vivo
Shared in product and in vivo
Shared in apheresis product and in vivo
Number of clonotypes
b
Number of TCR clonotypes in CAR+ cell
Pt10 37,952
Pt07 19,849
Pt06 17,932
Pt09 24,804
Pt03 24,003
Pt05 50,018
Pt04 24,676
Pt02 27,023
Pt12 10,015
Pt08 6,877
Pt01 10,675
Pt13 11,949
Pt11 13,646
Number of clonotypes
c
Number of TCR clonotypes in CAR- cell
Pt10 100,296
Pt07 62,695
Pt06 52,431
Pt09 57,999
Pt03 50,925
Pt05 68,733
Pt04 29,887
Pt02 19,944
Pt12 19,161
Pt08 12,775
Pt01 8,384
Pt13 17,706
Pt11 25,321
Number of clonotypes
d
Number of TCR clonotypes shared between CD4CD8-enriched apheresis and CAR+ T cells in product and CSF
CD4CD8 107297 3389 568
Product 3389 238634 1007
CSF 568 1007 37714
CD4CD8 Product CSF
Number of TCR clonotypes shared between CD4CD8-enriched apheresis and CAR- T cells in product and CSF
CD4CD8 107297 3430 3785
Product 3430 222109 3532
CSF 3785 3532 197147
CD4CD8 Product CSF
e
Patients with: 1 Product
⩾2 Products from different apheresis
2 Products from the same apheresis
f
Clonality
Apheresis: Pre-CAR Post-CAR
Pt03 Pt04 Pt05 Pt06 Pt07 Pt08 Pt09 Pt10 Pt11 Pt12 Pt13
g
Patients with: 1 Product
2 Products from same apheresis
⩾2 Products from different apheresis
Clonality
CAR+
CAR-
