# Supplementary material for: Anti-CAR Immunity Drives Acquired Therapeutic Resistance to GD2-CAR T Cell Therapy in Diffuse Midline Glioma
Source: medRxiv. 2026 Jul 9:2026.06.25.26356492. Preprint. [Version 1] doi: 10.64898/2026.06.25.26356492 (PMC13370534; doi:10.64898/2026.06.25.26356492)
Supplement: Supplementary file 5 [file media-5.pdf]

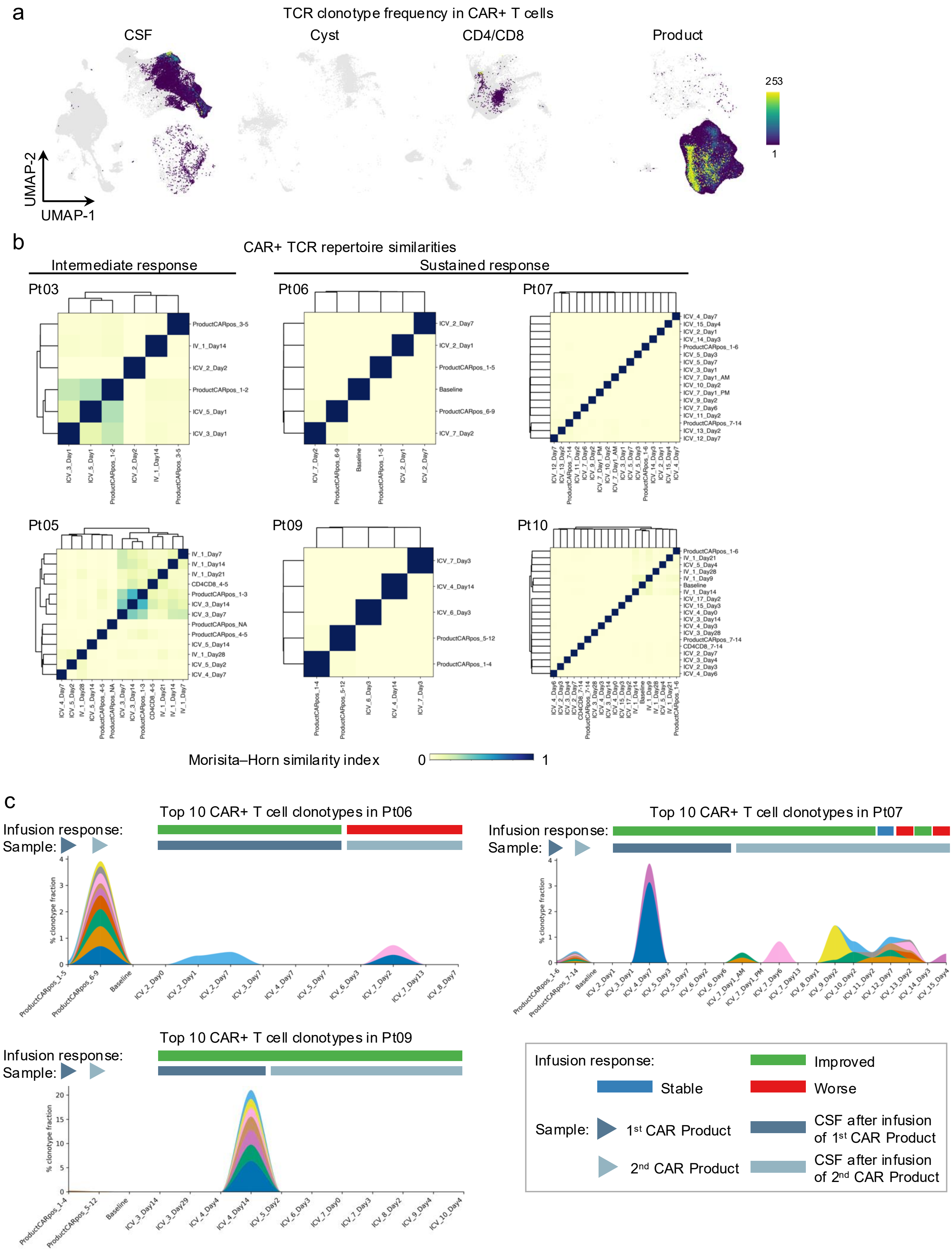

a
TCR clonotype frequency in CAR+ T cells
CSF
Cyst
CD4/CD8
Product
253
1
UMAP-2
UMAP-1
b
CAR+ TCR repertoire similarities
Intermediate response
Sustained response
Pt03
ProductCARpos_3-5
IV_1_Day14
ICV_2_Day2
ProductCARpos_1-2
ICV_5_Day1
ICV_3_Day1
Pt06
ICV_2_Day7
ICV_2_Day1
ProductCARpos_1-5
Baseline
ProductCARpos_6-9
ICV_7_Day2
Pt07
ICV_4_Day7
ICV_15_Day4
ICV_2_Day1
ICV_14_Day3
ProductCARpos_1-6
ICV_5_Day3
ICV_5_Day7
ICV_3_Day1
ICV_7_Day1_AM
ICV_10_Day2
ICV_7_Day1_PM
ICV_9_Day2
ICV_7_Day6
ICV_11_Day2
ProductCARpos_7-14
ICV_13_Day2
ICV_12_Day7
Pt05
IV_1_Day7
IV_1_Day14
IV_1_Day21
CD4CD8_4-5
ProductCARpos_1-3
ICV_3_Day14
ICV_3_Day7
ProductCARpos_NA
ProductCARpos_4-5
ICV_5_Day14
IV_1_Day28
ICV_5_Day2
ICV_4_Day7
Pt09
ICV_7_Day3
ICV_4_Day14
ICV_6_Day3
ProductCARpos_5-12
ProductCARpos_1-4
Pt10
ProductCARpos_1-6
IV_1_Day21
ICV_5_Day4
IV_1_Day28
IV_1_Day9
Baseline
IV_1_Day14
ICV_17_Day2
ICV_15_Day3
ICV_4_Day0
ICV_3_Day14
ICV_4_Day3
ICV_3_Day28
ProductCARpos_7-14
CD4CD8_7-14
ICV_2_Day7
ICV_3_Day4
ICV_2_Day3
ICV_4_Day6
Morisita–Horn similarity index
0
1
c
Top 10 CAR+ T cell clonotypes in Pt06
Infusion response:
Sample:
% clonotype fraction
ProductCARpos_1-5
ProductCARpos_6-9
Baseline
ICV_2_Day0
ICV_2_Day1
ICV_2_Day7
ICV_3_Day7
ICV_4_Day7
ICV_5_Day7
ICV_6_Day3
ICV_7_Day2
ICV_7_Day13
ICV_8_Day7
Top 10 CAR+ T cell clonotypes in Pt07
Infusion response:
Sample:
% clonotype fraction
ProductCARpos_1-6
ProductCARpos_7-14
Baseline
ICV_2_Day1
ICV_3_Day1
ICV_4_Day7
ICV_5_Day3
ICV_5_Day7
ICV_6_Day2
ICV_6_Day6
ICV_7_Day1_AM
ICV_7_Day1_PM
ICV_7_Day6
ICV_7_Day13
ICV_8_Day1
ICV_9_Day2
ICV_10_Day2
ICV_11_Day2
ICV_12_Day7
ICV_13_Day2
ICV_14_Day3
ICV_15_Day4
Top 10 CAR+ T cell clonotypes in Pt09
Infusion response:
Sample:
% clonotype fraction
ProductCARpos_1-4
ProductCARpos_5-12
Baseline
ICV_3_Day14
ICV_3_Day29
ICV_4_Day4
ICV_4_Day14
ICV_5_Day2
ICV_6_Day3
ICV_7_Day0
ICV_7_Day3
ICV_8_Day2
ICV_9_Day4
ICV_10_Day4
Infusion response:
Improved
Stable
Worse
Sample:
1st CAR Product
CSF after infusion of 1st CAR Product
2nd CAR Product
CSF after infusion of 2nd CAR Product
