# Supplementary material for: Anti-CAR Immunity Drives Acquired Therapeutic Resistance to GD2-CAR T Cell Therapy in Diffuse Midline Glioma
Source: medRxiv. 2026 Jul 9:2026.06.25.26356492. Preprint. [Version 1] doi: 10.64898/2026.06.25.26356492 (PMC13370534; doi:10.64898/2026.06.25.26356492)
Supplement: Supplementary file 6 [file media-6.pdf]

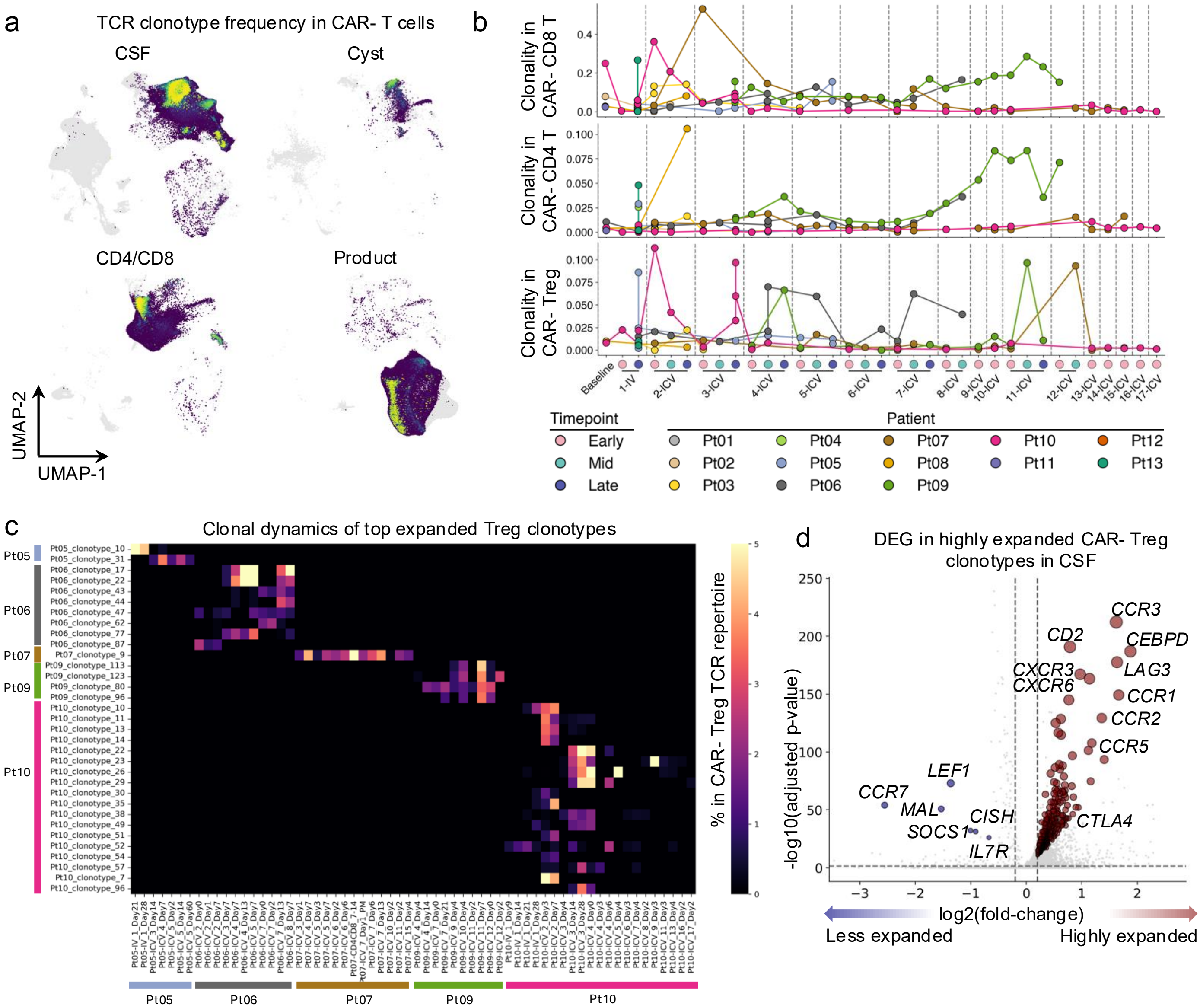

a
TCR clonotype frequency in CAR- T cells
CSF
Cyst
CD4/CD8
Product
UMAP-2
UMAP-1
b
Clonality in CAR- CD8 T
Clonality in CAR- CD4 T
Clonality in CAR- Treg
Timepoint
Early
Mid
Late
Patient
Pt01
Pt02
Pt03
Pt04
Pt05
Pt06
Pt07
Pt08
Pt09
Pt10
Pt11
Pt12
Pt13
c
Clonal dynamics of top expanded Treg clonotypes
% in CAR- Treg TCR repertoire
Pt05
Pt06
Pt07
Pt09
Pt10
d
DEG in highly expanded CAR- Treg clonotypes in CSF
-log10(adjusted p-value)
log2(fold-change)
Less expanded
Highly expanded
CCR3
CD2
CEBPD
LAG3
CXCR3
CXCR6
CCR1
CCR2
CCR5
LEF1
CCR7
MAL
CISH
SOCS1
IL7R
CTLA4
