# Supplementary material for: Anti-CAR Immunity Drives Acquired Therapeutic Resistance to GD2-CAR T Cell Therapy in Diffuse Midline Glioma
Source: medRxiv. 2026 Jul 9:2026.06.25.26356492. Preprint. [Version 1] doi: 10.64898/2026.06.25.26356492 (PMC13370534; doi:10.64898/2026.06.25.26356492)
Supplement: Supplementary file 7 [file media-7.pdf]

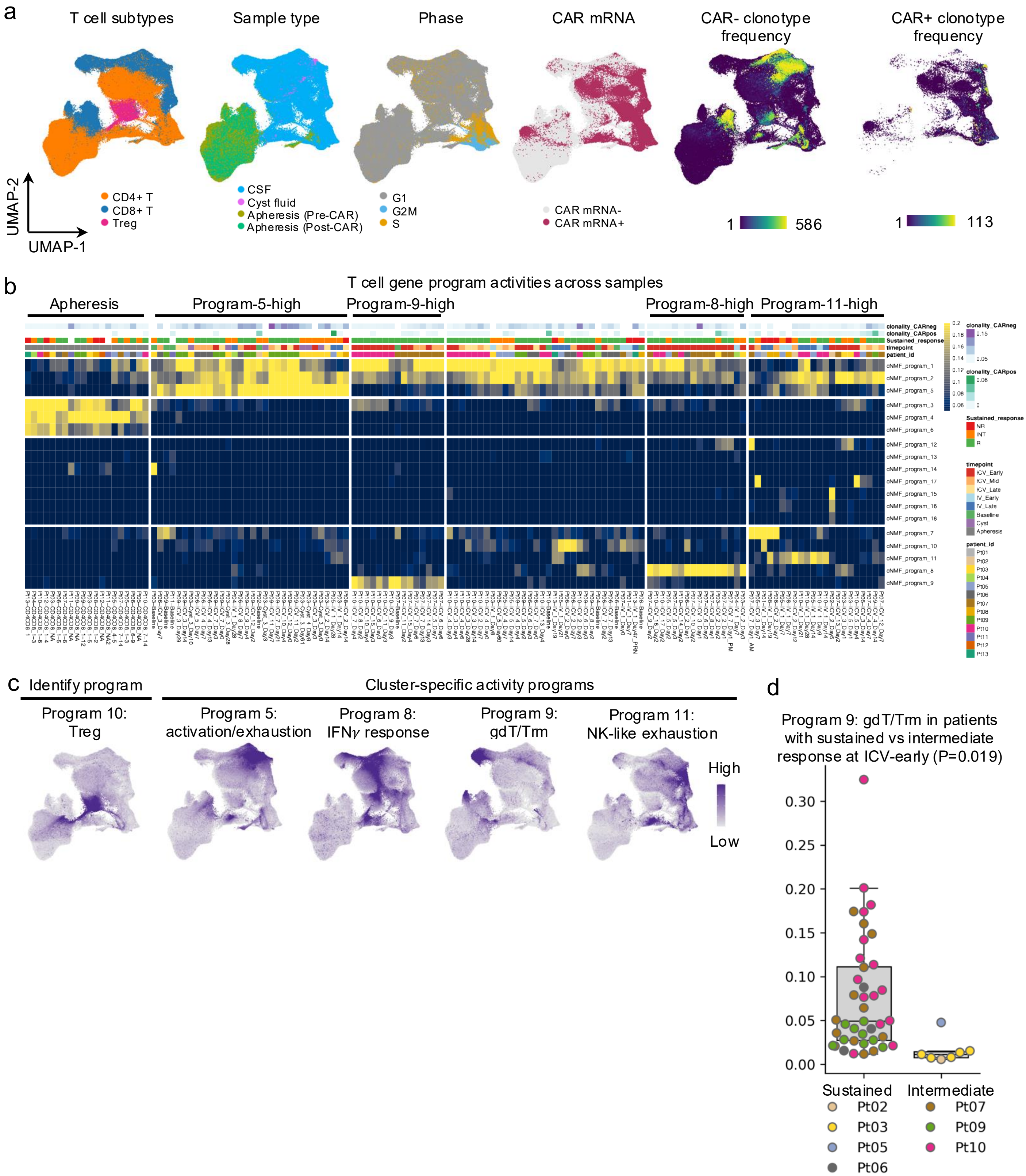
a
T cell subtypes
Sample type
Phase
CAR mRNA
CAR- clonotype frequency
CAR+ clonotype frequency
UMAP-2
UMAP-1
CD4+ T
CD8+ T
Treg
CSF
Cyst fluid
Apheresis (Pre-CAR)
Apheresis (Post-CAR)
G1
G2M
S
CAR mRNA-
CAR mRNA+
1
586
1
113
b
T cell gene program activities across samples
Apheresis
Program-5-high
Program-9-high
Program-8-high
Program-11-high
clonality_CARneg
clonality_CARpos
Sustained_response
timepoint
patient_id
cNMF_program_1
cNMF_program_2
cNMF_program_5
cNMF_program_3
cNMF_program_4
cNMF_program_6
cNMF_program_12
cNMF_program_13
cNMF_program_14
cNMF_program_17
cNMF_program_15
cNMF_program_16
cNMF_program_18
cNMF_program_7
cNMF_program_10
cNMF_program_11
cNMF_program_8
cNMF_program_9
Sustained_response
NR
INT
R
timepoint
ICV_Early
ICV_Mid
ICV_Late
IV_Early
IV_Late
Baseline
Cyst
Apheresis
patient_id
Pt01
Pt02
Pt03
Pt04
Pt05
Pt06
Pt07
Pt08
Pt09
Pt10
Pt11
Pt12
Pt13
c
Identify program
Cluster-specific activity programs
Program 10: Treg
Program 5: activation/exhaustion
Program 8: IFNγ response
Program 9: gdT/Trm
Program 11: NK-like exhaustion
High
Low
d
Program 9: gdT/Trm in patients with sustained vs intermediate response at ICV-early (P=0.019)
0.30
0.25
0.20
0.15
0.10
0.05
0.00
Sustained
Intermediate
Pt02
Pt03
Pt05
Pt06
Pt07
Pt09
Pt10
