# Supplementary material for: Anti-CAR Immunity Drives Acquired Therapeutic Resistance to GD2-CAR T Cell Therapy in Diffuse Midline Glioma
Source: medRxiv. 2026 Jul 9:2026.06.25.26356492. Preprint. [Version 1] doi: 10.64898/2026.06.25.26356492 (PMC13370534; doi:10.64898/2026.06.25.26356492)
Supplement: Supplementary file 8 [file media-8.pdf]

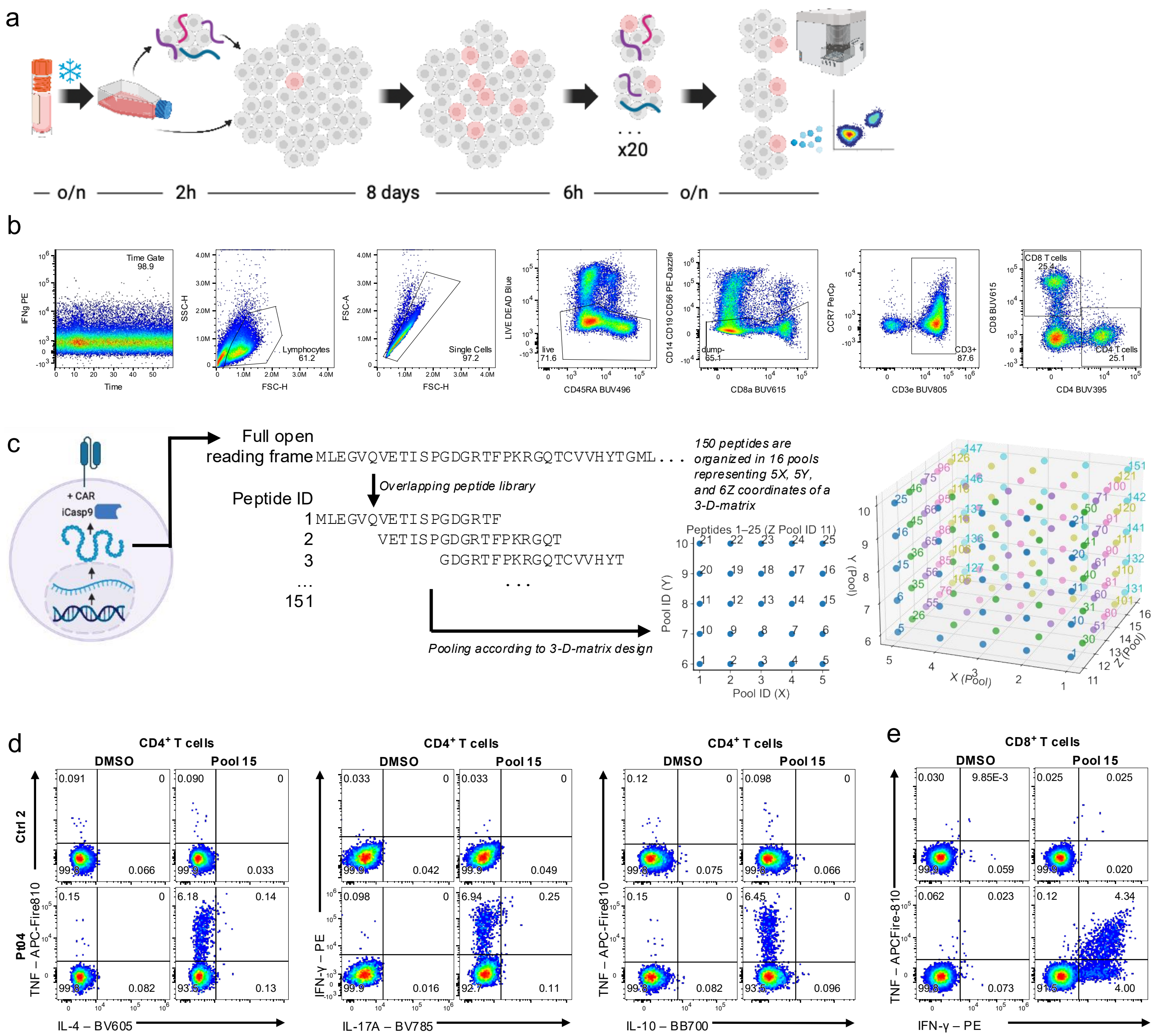
a
x20
o/n
2h
8 days
6h
o/n
b
Time Gate 98.9
IFNg PE
Time
SSC-H
FSC-H
Lymphocytes 61.2
FSC-A
Single Cells 97.2
LIVE DEAD Blue
live 71.6
CD45RA BUV496
CD14 CD19 CD56 PE-Dazzle
dump- 65.1
CD8a BUV615
CCR7 PerCp
CD3+ 87.6
CD3e BUV805
CD8 BUV615
CD8 T cells 25.4
CD4 T cells 25.1
CD4 BUV395
c
+ CAR
iCasp9
Full open reading frame MLEGVQVETISPGDGRTFPKRGQTCVVHYTGML...
Overlapping peptide library
Peptide ID
1 MLEGVQVETISPGDGRTF
2 VETISPGDGRTFPKRGQT
3 GDGRTFPKRGQTCVVHYT
...
151
Pooling according to 3-D-matrix design
150 peptides are organized in 16 pools representing 5X, 5Y, and 6Z coordinates of a 3-D-matrix
Peptides 1–25 (Z Pool ID 11)
Pool ID (Y)
Pool ID (X)
Y (Pool)
X (Pool)
Z (Pool)
d
CD4+ T cells
DMSO
Pool 15
Ctrl 2
Pt04
TNF – APC-Fire810
IL-4 – BV605
IFN-γ – PE
IL-17A – BV785
IL-10 – BB700
e
CD8+ T cells
TNF – APCFire-810
IFN-γ – PE
