# Supplementary material for: Anti-CAR Immunity Drives Acquired Therapeutic Resistance to GD2-CAR T Cell Therapy in Diffuse Midline Glioma
Source: medRxiv. 2026 Jul 9:2026.06.25.26356492. Preprint. [Version 1] doi: 10.64898/2026.06.25.26356492 (PMC13370534; doi:10.64898/2026.06.25.26356492)
Supplement: Supplementary file 9 [file media-9.pdf]

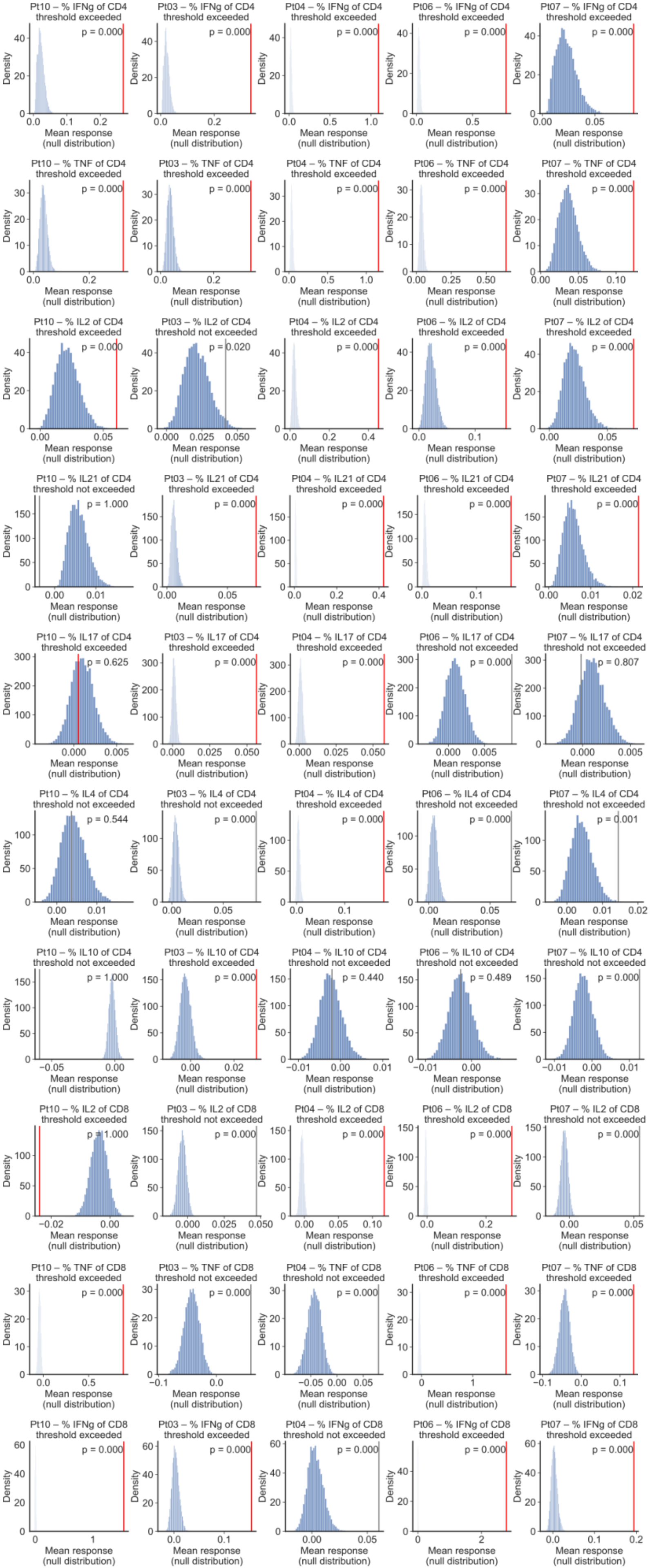

Pt10 – % IFNg of CD4
threshold exceeded
p = 0.000
Pt03 – % IFNg of CD4
threshold exceeded
p = 0.000
Pt04 – % IFNg of CD4
threshold exceeded
p = 0.000
Pt06 – % IFNg of CD4
threshold exceeded
p = 0.000
Pt07 – % IFNg of CD4
threshold exceeded
p = 0.000
Pt10 – % TNF of CD4
threshold exceeded
p = 0.000
Pt03 – % TNF of CD4
threshold exceeded
p = 0.000
Pt04 – % TNF of CD4
threshold exceeded
p = 0.000
Pt06 – % TNF of CD4
threshold exceeded
p = 0.000
Pt07 – % TNF of CD4
threshold exceeded
p = 0.000
Pt10 – % IL2 of CD4
threshold exceeded
p = 0.000
Pt03 – % IL2 of CD4
threshold not exceeded
p = 0.020
Pt04 – % IL2 of CD4
threshold exceeded
p = 0.000
Pt06 – % IL2 of CD4
threshold exceeded
p = 0.000
Pt07 – % IL2 of CD4
threshold exceeded
p = 0.000
Pt10 – % IL21 of CD4
threshold not exceeded
p = 1.000
Pt03 – % IL21 of CD4
threshold exceeded
p = 0.000
Pt04 – % IL21 of CD4
threshold exceeded
p = 0.000
Pt06 – % IL21 of CD4
threshold exceeded
p = 0.000
Pt07 – % IL21 of CD4
threshold exceeded
p = 0.000
Pt10 – % IL17 of CD4
threshold exceeded
p = 0.625
Pt03 – % IL17 of CD4
threshold exceeded
p = 0.000
Pt04 – % IL17 of CD4
threshold exceeded
p = 0.000
Pt06 – % IL17 of CD4
threshold not exceeded
p = 0.000
Pt07 – % IL17 of CD4
threshold not exceeded
p = 0.807
Pt10 – % IL4 of CD4
threshold not exceeded
p = 0.544
Pt03 – % IL4 of CD4
threshold not exceeded
p = 0.000
Pt04 – % IL4 of CD4
threshold exceeded
p = 0.000
Pt06 – % IL4 of CD4
threshold not exceeded
p = 0.000
Pt07 – % IL4 of CD4
threshold not exceeded
p = 0.001
Pt10 – % IL10 of CD4
threshold not exceeded
p = 1.000
Pt03 – % IL10 of CD4
threshold exceeded
p = 0.000
Pt04 – % IL10 of CD4
threshold not exceeded
p = 0.440
Pt06 – % IL10 of CD4
threshold not exceeded
p = 0.489
Pt07 – % IL10 of CD4
threshold not exceeded
p = 0.000
Pt10 – % IL2 of CD8
threshold exceeded
p = 1.000
Pt03 – % IL2 of CD8
threshold not exceeded
p = 0.000
Pt04 – % IL2 of CD8
threshold exceeded
p = 0.000
Pt06 – % IL2 of CD8
threshold exceeded
p = 0.000
Pt07 – % IL2 of CD8
threshold not exceeded
p = 0.000
Pt10 – % TNF of CD8
threshold exceeded
p = 0.000
Pt03 – % TNF of CD8
threshold not exceeded
p = 0.000
Pt04 – % TNF of CD8
threshold not exceeded
p = 0.000
Pt06 – % TNF of CD8
threshold exceeded
p = 0.000
Pt07 – % TNF of CD8
threshold exceeded
p = 0.000
Pt10 – % IFNg of CD8
threshold exceeded
p = 0.000
Pt03 – % IFNg of CD8
threshold exceeded
p = 0.000
Pt04 – % IFNg of CD8
threshold not exceeded
p = 0.000
Pt06 – % IFNg of CD8
threshold exceeded
p = 0.000
Pt07 – % IFNg of CD8
threshold exceeded
p = 0.000
Density
Mean response
(null distribution)
